# A modified hybrid hub-spoke model for assistive technology: institutional design for procurement, financing, and market-shaping

**DOI:** 10.3389/fresc.2026.1880845

**Published:** 2026-08-21

**Authors:** Goran Radosavljević, Aleksandra Lazevski

**Affiliations:** FEFA Faculty, Metropolitan University, Belgrade, Serbia

**Keywords:** assistive technology, hub-spoke model, institutional design, market-shaping, public policy, public procurement, supply chains

## Abstract

**Introduction:**

Access to assistive technology (AT) is often constrained by fragmented governance structures, inconsistent procurement mechanisms, and unequal regional capacities, resulting in gaps in service quality and limited equity for users. To address these systemic barriers, this paper introduces a modified hybrid hub-spoke model developed as an integrated organizational solution for optimizing AT delivery.

**Methods:**

The model is grounded in comparative insights from mature AT systems (Norway, Italy) and evolving systems (Armenia, Ukraine), as well as an applied feasibility study conducted in Serbia. It combines the efficiency and standardization benefits of a central hub, responsible for procurement, quality assurance, training, advanced assessment, data systems, and refurbishment, with the accessibility and user-centred support provided by regional spoke centres. These centres deliver local evaluation, maintenance, follow-up, and everyday user assistance, ensuring that service quality remains consistent regardless of geographic location.

**Results:**

The model is designed to enhance stakeholder coordination, strengthen supply chains, support transparent catalogue-based procurement, generate cost savings through reuse and refurbishment, and reduce territorial disparities. By articulating the operational, financial, and market-shaping mechanisms of the model, the paper positions it as a scalable and transferable framework applicable across diverse contexts.

**Discussion:**

The modified hybrid hub-spoke model offers a viable pathway toward more equitable, efficient, and sustainable AT provision systems, of direct benefit to AT users, particularly children and adults, and of practical relevance to ministries, resource centres, and international partners responsible for the design of such systems.

## 1 Introduction

The 2022 Global Report on Assistive Technology shows that more than 2.5 billion people globally need at least one assistive product, while close to one billion have unmet needs (1). In some low- and middle-income countries (LMICs), coverage rates are reported as low as three per cent, while in the WHO European Region between 65 and 80 per cent of those who need AT lack access (2, 3). The gap is not primarily a shortage of resources. It is how AT delivery is organised: who procures and finances AT, who assesses needs, who maintains and replaces equipment, and how all these functions are coordinated across sectors.

Two structural features of the AT landscape further constrain access. First, supply chains are fragmented and concentrated. Production is geographically clustered in a small number of high-income economies, leaving many countries dependent on imports, donations, or low-quality domestic substitutes that are rarely aligned with population needs (4). Tariffs, customs procedures, value-added tax treatment, and the absence of dedicated tariff classifications in the Harmonized System raise effective prices and limit the volumes that public buyers can afford. Second, demand is fragmented institutionally. AT users typically interact with multiple ministries, health, social protection, education, employment, etc., each with its own catalogue, eligibility rules, and procurement procedures. The result is a thin and discontinuous market in which suppliers face high transaction costs, public buyers cannot aggregate volumes, and users cannot obtain the products they need.

Global health has developed a deliberate response to these problems, known as market-shaping. In simple terms, market-shaping refers to a set of coordinated actions by public buyers to make the market for a product work better, improving availability, reducing prices, and raising quality, rather than leaving these outcomes to chance. Instead of treating fragmented supply, high prices, and uneven quality as fixed market conditions, market-shaping interventions actively reshape how markets function to improve availability, affordability, and quality of essential health products (5, 6). The main instruments are by now well known, although their application to assistive products is still underdeveloped. Long-term volume commitments give suppliers the certainty to invest in products. Transparent price disclosure prevents wide price differences between buyers of the same product. Demand aggregation combines fragmented orders from many buyers into a single large one, which strengthens negotiating power. Harmonised technical specifications reduce the number of products variants that manufacturers need to produce., and risk-sharing arrangements divide uncertainty between buyers and suppliers (5). The 2018 GREAT summit identified procurement and supply systems as “among the most consequential leverage points for system-wide change in AT” (7, p. 458). However, recent comparative work on AT financing makes clear that these instruments cannot be transplanted in isolation. According to Tay-Teo et al. (7), the choice of financing arrangement (pooled public budgets, social insurance, vouchers, or out-of-pocket payment) directly determines whether market-shaping is feasible at all.

Within national systems, the institutional architecture through which AT is delivered determines whether market-shaping instruments can in fact be deployed. Centralised systems concentrate procurement, ownership, refurbishment, and quality assurance at the national level, as in Norway's NAV-led model (8). Decentralised systems distribute these functions across regional or municipal authorities, often relying on partnerships with non-governmental organisations and on framework legislation that delegates implementation to subnational levels, as in much of Italy and the Nordic municipal systems (9, 10). Each pure form has well-documented strengths and weaknesses. Centralised systems achieve standardisation, transparent procurement, and economies of scale, but require strong administrative capacity and risk being remote from users. Decentralised systems are responsive to local needs but produce uneven coverage, fragmented information systems, and duplication of effort, particularly in countries with significant territorial inequalities (11, 12).

Hub-spoke arrangements have emerged in healthcare and social services as an attempt to combine the efficiency of central coordination with the accessibility of distributed service delivery (13, 14). In its conventional form, however, the hub-spoke model treats the central hub primarily as a clinical or technical reference centre and the spokes as service outlets. Operations-research formulations of hub-spoke design problems likewise emphasise routing efficiency rather than market-shaping or supply chain governance (15). When applied to AT, this framing leaves several functions weakly specified. How are procurement and the AT catalogue governed? How are device ownership and refurbishment loops organised? How is operational financing divided between central and local budgets? And how does the system shape the upstream market for assistive products? These questions matter, because the cost-effectiveness and equity of AT systems depend heavily on precisely these dimensions (1).

In this paper we develop a modified hybrid hub-spoke model that addresses these gaps and positions national AT delivery as an instrument of market-shaping. The “hybrid” qualifier refers to a deliberate blending of centralised and decentralised functions rather than a default to one extreme. The “modification” refers to four explicit additions relative to the conventional hub-spoke template: (i) integration of a refurbishment and reuse loop within the hub's operational scope; (ii) a catalogue- and framework-agreement-based procurement mechanism that doubles as a market-shaping instrument; (iii) a graduated assessment architecture in which spokes perform routine evaluation while the hub handles complex and rare cases; and (iv) a financial architecture that distinguishes operational financing of the network from device-level financing, accommodating both budget-based and grant-based options. The model is derived from comparative analysis of four European AT systems, two mature (Norway and Italy) and two evolving (Armenia and Ukraine). Model is also tested in an applied feasibility study in Serbia, where it was developed as the organisational and financial blueprint for a future National Centre for Assistive Technologies and a network of Resource Centres.

The paper's principal contribution is therefore a normative institutional design, derived inductively from four European cases and tested for feasibility rather than for implementation outcomes. Where the argument draws on evidence from existing systems, that evidence is identified as such. Where the model's expected effects follow from its logic but have not been directly demonstrated, they are identified as predictions the model generates.

## 2 Methods

The study employs a multi-method design that combines purposive comparative case analysis with an applied feasibility study. Our objective was to identify recurrent organisational features of AT delivery systems that produce equitable and efficient outcomes and that activate market-shaping levers, and then to translate those features into a coherent and transferable model that can be tested in a transitioning context. The research was conducted between 2023 and 2025 in support of the AT system development process led by the Ministry of Education of the Republic of Serbia and UNICEF Serbia.

### 2.1 Comparative case selection

We selected four cases purposively to maximise variance along two dimensions: how developed the AT system is (mature or evolving), and how AT provision is organised across levels of government (centralised or decentralised). Among mature systems, Norway represents the most fully developed centralised model in Europe, organised around the Norwegian Labour and Welfare Administration (NAV), with sixteen Assistive Technology Centres, a centralised procurement unit, and a well-established refurbishment programme (8). Italy represents a decentralised model with strong regional variation. We examined the Emilia-Romagna region in detail because of its long-standing partnership between the regional health service and AIAS Bologna onlus, a non-governmental organisation operating the Regional Centre for Assistive Technology (9, 16). Among evolving systems, Armenia is in transition from a multi-ministerial provision system toward an organised network of resource centres, with twenty Regional Pedagogical-Psychological Support Centres operating as territorial nodes (16). Ukraine illustrates the challenges of fragmented, multi-ministerial provision in which the Medical and Social Expert Commission, the Ministry of Social Policy, the Ministry of Health, and the Ministry of Education each exercise partial authority over distinct AT categories (17). The mature-evolving and centralised-decentralised contrasts allow us to conduct a structured comparison rather than a most-similar or most-different design alone.

### 2.2 Analytical framework

We mapped each case using a six-step framework adapted from the WHO and UNICEF (1) AT access pathway. The original pathway is user centred. For the purposes of organisational analysis, we adapted it in two respects. First, we shifted the perspective from the individual user to the institution, to describe who holds responsibility for each step rather than what happens to a user. Second, we added two cross-cutting dimensions, place of use (home, school, workplace) and source of funding. The resulting framework distinguishes three stages, setup and organisation, awareness and coordination, provision and realisation, each disaggregated into discrete steps such as catalogue management, procurement, assessment, training, follow-up, and review. To this scaffold we added a market-shaping lens. Each case was further mapped against five instruments drawn from the global health market-shaping literature (5, 6): demand aggregation, transparent price discovery, technical specification harmonisation, multi-year commitments, and supply diversification. This allowed us to identify which instruments each system activates and through what institutional means.

### 2.3 Data sources

Data for the comparative cases were collected from three sources. First, we conducted a systematic review of legislation and policy documents in each country. Second we drew on secondary analyses published by the European Agency for Special Needs and Inclusive Education (18), the Swedish Agency for Participation (19), the Association for the Advancement of Assistive Technology in Europe (10), and comparative European studies on AT access and service delivery (1, 2, 20). Third, we conducted five semi-structured expert interviews online, between August and September 2023. In Norway, we interviewed Tone Øderud (SINTEF) on 9 August 2023 and Berit Stølen (NAV) on 11 August 2023. In Ukraine, we interviewed Lesya Kalandyak (Momentum Wheel for Humanity) on 1 August 2023 and Hanna Usatenko (AEC Community) on 11 August 2023. In Armenia, we interviewed Maya Simonyan (UNICEF Armenia) on 22 September 2023. Each interview lasted up to 60 min. Interviews were conducted by the authors and followed a written interview guide organised around six topics: legal framework, institutional organisation and division of responsibility, service delivery process, funding scheme, current challenges, and lessons transferable to a transitioning national setting. All participants were interviewed in their institutional capacity and gave verbal informed consent at the start of each interview to be recorded, to be identified by name, and to have the interview material used in the resulting research outputs. Interviews were audio-recorded on that basis, and notes were produced from the recordings for thematic coding. Participants were identified purposively on the basis of their operational responsibility for AT provision at the national level and were recruited through the UNICEF Europe and Central Asia Regional Office network and through the authors’ prior professional contacts in the AT community. For Italy, the equivalent primary material was obtained through a project study visit to Bologna in the Emilia-Romagna region in 2023, during which the authors met representatives of AIAS Bologna onlus and the Local Health Trust of Bologna and discussed the operational and financial arrangements of the regional AT provision model in detail.

The Serbian feasibility study drew on a further set of interviews and consultations conducted in 2023 and 2024. These included interviews with representatives of the Ministry of Education of the Republic of Serbia responsible for AT in the education sector, the directors of the Resource Centres in Pančevo and Novi Sad, and the national and international experts engaged on the underlying project team. A small case study of cooperation between the City of Pančevo and the “Mara Mandić” Resource Centre provided a concrete illustration of how AT provision currently operates at the interface between local self-government, the Resource Centre, and the Intersectoral Committee. This material informed the situation analysis reported in Section 3.3.1 and the operational and financial design proposals developed in Sections 3.3.2 and Sections 3.3.3.

### 2.4 Synthesis and model construction

Cross-case synthesis followed an inductive logic. For each of the four cases, the documentary sources, the AAATE and Nordic comparative studies, and the interview material were first coded independently against the six-step organisational framework and the five market-shaping instruments described in Section 2.2. We then compared the resulting profiles across cases to identify functional patterns that recurred in the systems that achieved cost efficiency, equity, and market-shaping leverage, and were absent in those that did not. Four such patterns emerged: centralised catalogue and procurement, distributed assessment with referral pathways for complex cases, ownership-based refurbishment, and multi-source financing. We assembled recurrent functional patterns identified in the comparative analysis (centralised catalogue and procurement, distributed assessment with referral pathways for complex cases, ownership-based refurbishment, and multi-source financing) These were assembled into the modified hybrid hub-spoke model presented in Section 3.2. The model was than operationalised for Serbia through institutional design proposals reported in Section 3.3., drawing on parameters and benchmarks from the comparator systems. Detailed cost projections were prepared as part of the feasibility work for UNICEF Serbia but are not reproduced here, as this paper's focus is on institutional design rather than on financial analysis. The applied stage thus served both as a test of feasibility in a transitioning context and as an opportunity to refine the model considering implementation constraints.

## 3 Results

### 3.1 Comparative analysis of AT delivery systems

The four cases differ along almost every dimension: legal basis, lead institution, procurement architecture, financing logic, and the role of civil society. However, they converge on a small set of structural choices that recur in successful arrangements. We present the cases in turn, with explicit attention to procurement, catalogue practices, supply chains, and refurbishment, before drawing out the cross-case patterns.

#### 3.1.1 Mature systems: Norway and Italy

Norwegian AT provision is organised around the National Insurance Scheme and delivered through NAV, which combines policy stewardship, centralised procurement, and operational coordination with sixteen regional Assistive Technology Centres. The legal basis is the National Insurance Act, supplemented by the Working Environment Act and the Education Act, with the UN Convention on the Rights of Persons with Disabilities incorporated through a 2013 ratification (21).

The defining feature of the Norwegian system from a market-shaping perspective is centralised procurement. The NAV Central Unit of Purchases concludes framework agreements with vendors, typically for two years and extendable to four, covering all major categories of assistive devices (8). Procurement is preceded by structured tender procedures of twelve to eighteen months and follows the Public Procurement Act, including European-level publication. Demand aggregation thus operates at national scale, technical specifications are harmonised through the catalogue, and multi-year commitments give suppliers planning horizons that translate into more competitive pricing. According to Stenberg et al. (20), the Norwegian arrangement is among the most consolidated in Europe.

The second defining feature is ownership. NAV retains ownership of the assistive devices issued to users; users effectively borrow devices, which return to ATCs at the end of use. This ownership structure underpins a systematic refurbishment programme that, according to Sund (8), accounts for approximately 30% of the AT budget. By way of comparison, refurbishment in Ukraine is reported at 2%–3% of total AT expenditure (17). The Norwegian arrangement is consequential for the upstream market in two ways. First, it internalises a portion of the device life cycle within the public system, redirecting demand from new acquisition toward maintenance and parts and creating a service market that domestic firms can credibly enter. Second, it limits the volumes of new devices that flow through the primary procurement channel, which sharpens price competition for the remaining tenders. The total AT-device cost in 2022 was approximately €300 million, supplemented by ATC operating budgets covering staffing, logistics, training, and stock; one mid-sized ATC reported an annual operating budget of €5 million (8).

The Norwegian system also relies on a graduated case-management logic. Routine cases are handled at the local NAV office and the municipality, more complex cases at the ATC, and rare or specialist cases at the central NAV unit. The challenges identified by Norwegian respondents are informative for transfer purposes. They include difficulties in follow-up and in monitoring whether devices are used, the rising relative weight of the elderly population (which crowds out attention to children), and persistent gaps in municipal staff training (8).

In Italy, AT provision is anchored in the National Health System and complemented by sector-specific regulation for employment and education. The principal legal references are Framework Law 104/92, Ministerial Decree 332/99, Law 68/99 on the rights of disabled persons in employment, and Law 4/2004 on e-accessibility (9). Regional health authorities operate within national framework legislation but enjoy substantial autonomy, which produces significant inter-regional variation. A national catalogue, the Nomenclatore Tariffario, lists admissible products and reimbursement levels, performing the technical specification function nationally even where procurement is regional.

The Emilia-Romagna region offers an instructive sub-case because of its long-standing arrangement between the regional health trust and AIAS Bologna onlus, a non-governmental organisation that has operated the Regional Centre for Assistive Technology since 2000. Two financing mechanisms structure the Emilia-Romagna model. First, AIAS receives a contractual lump sum from the Local Health Trust of Bologna, supplemented since 2019 by unit-cost reimbursement against four defined service groups, each with a minimum service threshold (22). Second, since 2017 a national funding stream has been allocated on a triennial project basis for AT for pupils and students with disabilities. Projects are evaluated by a regional commission, ranked at provincial level, and implemented through Territorial Support Centres operating in cooperation with schools. The Territorial Support Centre network, ninety-two centres distributed across the country, provides a uniform educational interface that complements the heterogeneous health-sector arrangements (9). From a market-shaping perspective, the Italian configuration combines two compensating mechanisms: a national catalogue that harmonises specifications across regions, and a service-level partnership architecture that allows non-governmental specialisation while retaining public accountability through unit-cost contracts. It also illustrates the limits of decentralisation, as services for AT not listed in the national catalogue must be paid out-of-pocket, with reimbursement available only through partial regional or fiscal schemes.

#### 3.1.2 Evolving systems: Armenia and Ukraine

Armenia's AT system is currently being restructured under the 2021 Law on the Rights of Persons with Disabilities and parallel amendments to the Law on Foundations of Education (16). Three ministries, Labour and Social Affairs, Education, Science, Culture and Sport, and Health, share responsibility, with the Ministry of Labour and Social Affairs holding the dominant role for disability assessment, certification, and provision of mobility, prosthetic, and hearing devices. Approximately 12,000–13,000 AT devices are issued annually to between 5,000 and 7,000 individuals from the state budget. A national AT list has been developed and is being progressively expanded, technical specifications are being revised to improve quality, and an assessment instrument grounded in the International Classification of Functioning, Disability and Health has been in force since 2023 (16).

The most consequential ongoing reform is the conversion of seven remaining specialised schools into Regional Pedagogical-Psychological Support Centres, complementing twenty existing centres. The transformation logic, using the existing physical infrastructure, staff, and regional embeddedness of special schools as the basis for a new network of resource centres, is the same approach we later propose for Serbia (23). From a market-shaping standpoint, Armenia is in the early stages of activating its instruments. The AT list provides specification harmonisation, but demand aggregation across ministries remains incomplete, and multi-year commitments to suppliers are constrained by annual budget cycles. In our view, the Armenian case shows that even in fiscally constrained settings, an organised network can be assembled by repurposing existing institutions, provided that legislation, AT lists, and assessment standards are aligned.

Ukrainian provision is organised through a state programme of rehabilitation under the Law on Rehabilitation of Persons with Disability, and a series of cabinet resolutions and ministerial orders (23). Responsibility is fragmented across the Ministry of Social Policy (lead), Ministry of Health, Ministry of Education and Science, Ministry of Youth and Sports, and Ministry of Culture, with the Ukrainian Research Institute for Prosthetics and Rehabilitation playing an influential technical role. Eligibility is mediated by the Medical and Social Expert Commission, which both certifies disability status and prescribes the individual rehabilitation programme that determines AT entitlements. The wartime context has further complicated provision; recent assessments by the WHO Regional Office for Europe document substantial pressure on rehabilitation services and supply chains (24).

The Ukrainian case is instructive precisely because it illustrates the costs of unresolved fragmentation. Because the three principal ministries develop parallel rather than joint procedures, demand cannot be aggregated across the system; suppliers face multiple smaller tenders rather than a single national one, with predictable consequences for prices and quality. The procurement architecture relies on individual contracts between users, suppliers, and local social protection departments rather than on aggregated framework agreements. Refurbishment is marginal at 2%–3% of expenditure, partly because device ownership rests with users rather than the system, which forecloses systematic reuse. Repair and maintenance budgets are reported as insufficient. Recent reforms include an electronic cabinet for service applications and a pilot online screening instrument for early childhood, but these address the user-interface layer rather than the underlying coordination and market problem. In other words, digital interfaces do not by themselves resolve institutional fragmentation.

#### 3.1.3 Cross-case structural patterns

Three structural patterns recur across the four cases, and we use them to construct the modified hybrid hub-spoke model presented in Section 3.2. First, the cases that achieve cost efficiency, equity, and market-shaping leverage (Norway, and to a lesser extent Emilia-Romagna) do so through a specific combination of catalogue-based procurement, framework agreements with vendors, and system ownership of devices. The combination matters. Catalogues without aggregated procurement do not unlock economies of scale, while procurement without ownership forecloses systematic refurbishment. Second, distributed service delivery succeeds when paired with an explicit referral architecture between routine cases handled locally and complex cases handled centrally. This architecture is most explicit in the Norwegian system. Third, fragmented multi-ministerial governance, which we observe most clearly in Ukraine, but which also visibly constrains Armenia, imposes coordination costs that no amount of investment in service delivery can fully compensate, and decisively undermines the ability to activate market-shaping instruments. The lesson here is not that AT must reside under a single ministry, but that whichever institution leads must hold operational authority across procurement, catalogue management, and standards-setting. These three patterns inform each component of the model that follows.

### 3.2 The modified hybrid hub-spoke model

The modified hybrid hub-spoke model is a normative design synthesised from our comparative analysis. We call it hybrid because it deliberately distributes functions between a central hub and regional spokes, instead of defaulting to either centralisation or decentralisation. We call it modified because it adds four features to the conventional hub-spoke template: an integrated refurbishment and reuse loop, a catalogue- and framework-agreement-based procurement mechanism, a graduated assessment architecture, and a dual-track financial architecture distinguishing operational from device-level financing. Together, these features define three operating mechanisms (operational, financial, and market-shaping), with implications for trade and supply chains discussed at the end of this section.

#### 3.2.1 Conceptual foundation

The model rests on three premises drawn from the comparative evidence. First, equity of access in territorially heterogeneous countries cannot be sustained by purely local financing of AT devices, because municipal fiscal capacity correlates strongly with development indicators that are themselves correlated with disability prevalence. Centralised financing of devices, or at minimum centralised pooling, is therefore a precondition for equity. The decentralisation literature has long noted that subnational delivery without compensating central financing tends to amplify rather than reduce horizontal inequalities (11, 12, 25).

Second, the technical complexity of AT assessment varies by case. Most assessments can be performed competently at regional level, but a non-trivial minority require specialist knowledge, advanced equipment, or rare combinations of expertise. A two-tier assessment architecture aligns specialist capacity with specialist demand, paralleling the integrated practice unit logic developed in healthcare strategy more broadly (14, 26).

Third, AT systems are also markets. The way a public system procures, lists, and replaces devices shapes the upstream supply market, domestic manufacturers, importers, and service providers. We see this market-shaping role as integral to the model, not as a side-effect, and we follow established practice in adjacent areas of global health where market-shaping has proven decisive for access at scale (5–7).

#### 3.2.2 Architecture: hub and spokes

**Figure 1:**
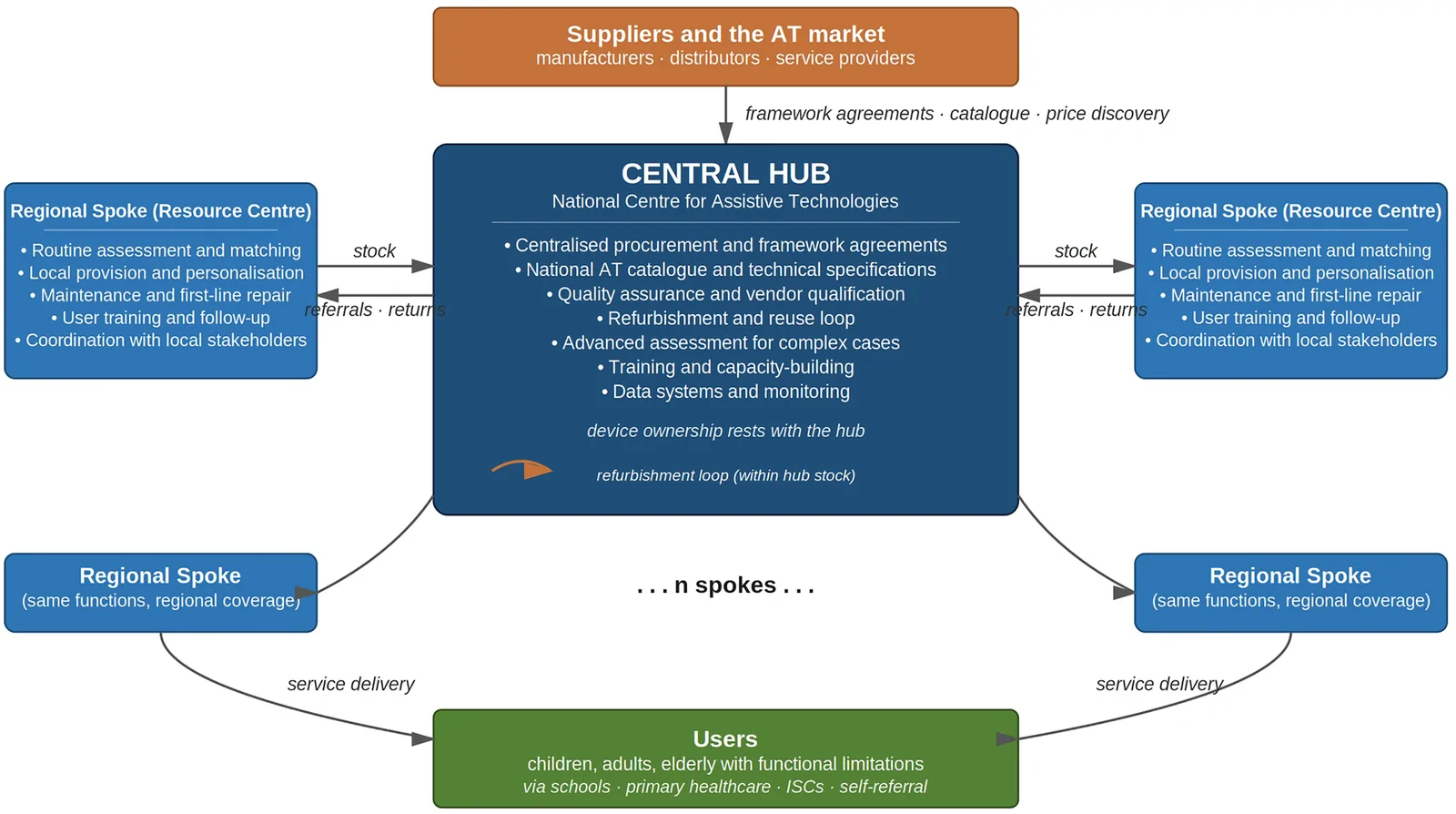
Architecture of the modified hybrid hub-spoke model. The central hub holds market-facing functions (procurement, catalogue, ownership, refurbishment) together with quality assurance, training, advanced assessment, and data systems. Regional spokes hold user-facing functions (routine assessment, provision and personalisation, maintenance, training, and follow-up) and coordinate with local stakeholders. Bidirectional flows between hub and spokes carry stock requisitions, complex referrals, and device returns. The hub interfaces with the upstream market through framework agreements and the catalogue, and with users indirectly through the spokes.

The model has a single national hub and a network of regional spokes, with explicit functional separation between them (Figure 1). The hub is the system's point of contact with the market and with national policy; the spokes are its point of contact with users. The architecture is deliberately asymmetric. Market-facing functions (procurement, catalogue, ownership) are concentrated at the hub to capture economies of scale and exercise market-shaping leverage, while user-facing functions (assessment, fitting, follow-up, repair) are distributed across the spokes to maintain proximity and responsiveness.

The hub is conceived as a single national institution holding seven functions:

- Centralised procurement governing the entire device portfolio for the network through framework agreements concluded after competitive tender, modelled on the Norwegian NAV Central Unit of Purchases.
- Catalogue management defining and maintaining the national AT list, the technical specifications attached to each entry, and the ranking of equivalent products. The catalogue serves simultaneously as an internal procurement tool and an external signal to the market.
- Quality assurance covering technical conformity testing, vendor qualification, and post-market surveillance.
- Advanced assessment and consultation providing specialist input on rare and complex cases referred upward by the spokes.
- Training and capacity-building delivering continuing education for spoke staff, municipal social workers, and educators, including the methodological standards under which the spokes operate.
- Data systems and monitoring maintaining a unified registry of users, devices, assessments, and outcomes, which supports planning, accountability, and longitudinal evaluation.
- Refurbishment and reuse, the most distinctive of the modifications, treated separately within the operational mechanism.

The spokes operate as regional one-stop service points close to users. Their functions are designed to complement the hub, not to duplicate it. Routine assessment establishes user needs and matches them with catalogue products, with onward referral to the hub for cases beyond regional competence. Local provision and fitting issues devices from regional stock, including the personalisation and adaptation that often accompanies issuance. Maintenance and repair maintain a first-line technical capability, including for electromedical devices that require periodic functionality checks. User training equips users, family members, and front-line professionals to use devices effectively. Follow-up tracks device use identifies abandonment or misfit, and triggers replacement, refurbishment return, or repair. Coordination with local stakeholders, Intersectoral Committees, schools, primary healthcare, and social work centres, integrates AT provision into local service ecosystems.

What distinguishes our architecture from a canonical hub-spoke arrangement, beyond the four additions listed above, is the explicit treatment of the hub-spoke interface as a governance question and not just a logistics one. The strategic-management literature on hub-and-spoke arrangements describes two contrasting approaches (27). In one, the central hub imposes decisions on the regional units. In the other, the hub and the regional units negotiate jointly across a broad agenda. Our architecture combines both. For a limited set of issues, procurement, the catalogue, and technical standards, the hub decides. For broader issues, such as service mix, training priorities, and local coordination, decisions are negotiated with the spokes. This division mirrors the operational logic of integrated practice units in health care, where centralised functions reinforce rather than substitute for distributed ones (14).

#### 3.2.3 Operating mechanisms

We describe how the architecture functions in practice through three mechanisms. The operational mechanism describes how cases flow through the system; the financial mechanism describes how the system is funded; and the market-shaping mechanism describes how the system's purchasing behaviour shapes the upstream market for assistive products.

##### 3.2.3.1 Operational mechanism

**Figure 2:**
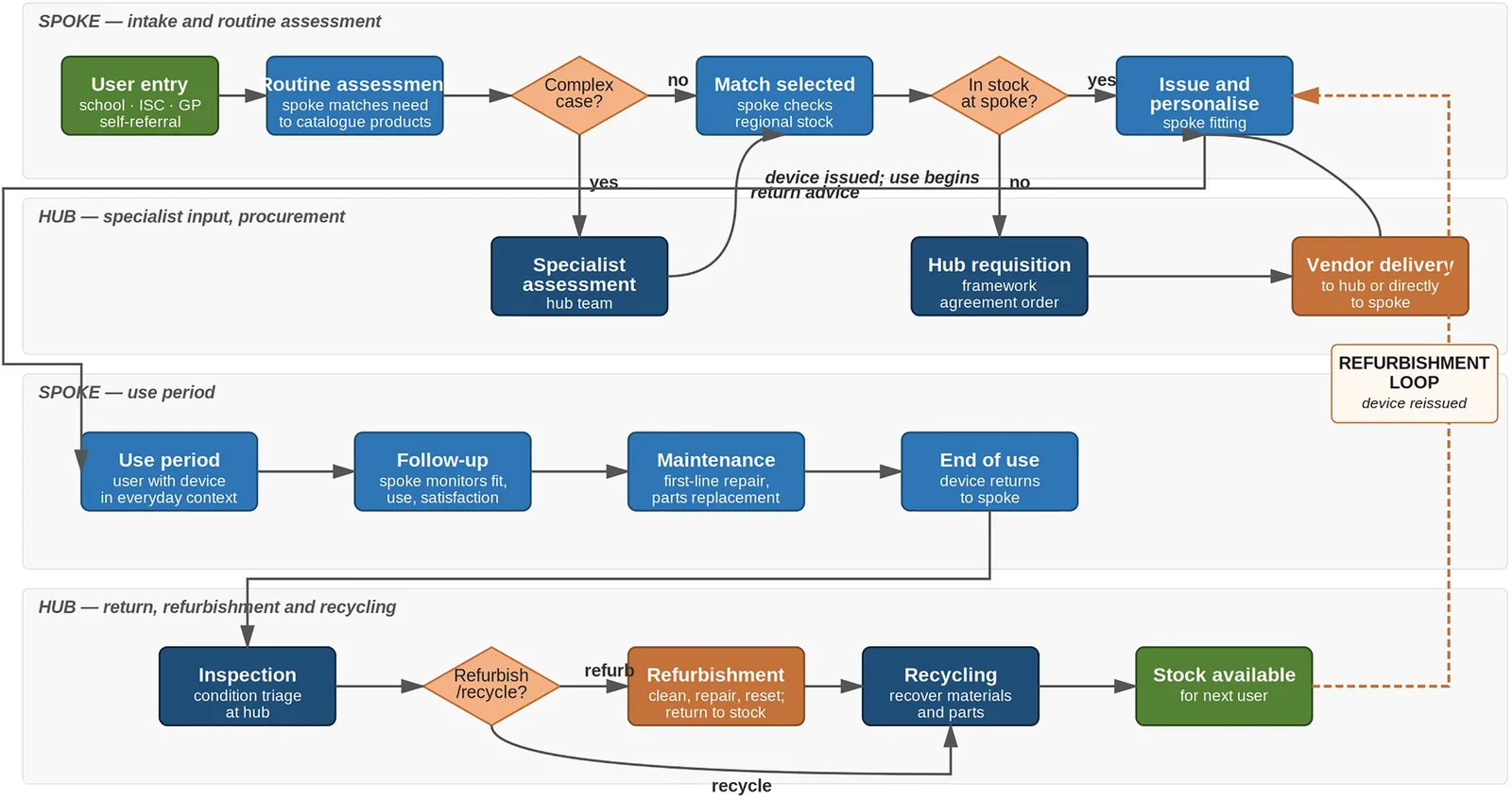
AT provision process flow with the refurbishment loop. AT provision process flow with the refurbishment loop. The figure presents the case life cycle as a swim-lane diagram across spoke (regional), hub (national), and supplier lanes. Routine assessment and matching occur at the spoke; complex cases are referred upward to the hub specialist team and returned with advice. If the matched device is not in regional stock, the spoke requisitions it through the hub against the framework agreement; vendor delivery follows. After issuance, the spoke conducts follow-up, maintenance, and end-of-use return. Returned devices are inspected and either refurbished, recycled, or reissued; the dashed orange path indicates the refurbishment loop, which returns devices to usable stock for the next user.

The operational mechanism is illustrated in Figure 2. Users enter through the spoke, normally on referral from a school, a primary healthcare provider, an Intersectoral Committee, or a self-referral via an electronic interface. Routine assessment and matching occur at the spoke. If the assessment requires specialist input, the case is referred upward to the hub, but the spoke retains relationship management. Once a device is matched, the spoke checks regional stock. If the device is available, it is issued and personalised. If it is unavailable, the spoke requisitions it from the hub against the national framework agreement, and the hub arranges delivery to the spoke. Throughout the use period the spoke maintains follow-up; at the end of use, devices return to the spoke, are inspected, and either reissued, refurbished, or recycled.

Refurbishment is the operational hinge that links use cycles into a stock-management system rather than a one-shot issuance, and it is one of the most consequential features of the model. The refurbishment loop, marked in dashed orange in Figure 2, returns inspected devices to usable stock through cleaning, repair, parts replacement, and resetting, after which they re-enter the issuance flow for the next user. Devices unsuitable for refurbishment are recycled for parts and materials. According to Sund (8), ownership-based refurbishment in Norway accounts for 30% of AT expenditure in steady state, which generates substantial fiscal space without compromising user outcomes. The operational viability of the loop depends on system ownership of devices. When devices are owned by users, as in Ukraine, returns and refurbishment are not enforceable, and the loop cannot be activated.

This ownership arrangement does not diminish the user's position. Drawing on the Norwegian model, the user is fully involved in selecting the device that fits their needs and receives it free of charge but does not own it. A device is returned only when it is no longer needed, for example when a child has outgrown it or the user's condition has changed, at which point it can be refurbished and reissued to someone else. Return therefore coincides with the end of need rather than interrupting access, and a user whose needs change again is reassessed and reissued in the normal way. This is what allows devices to be provided free of charge and prevents serviceable products from being discarded after a single use.

##### 3.2.3.2 Financial mechanism

The financial mechanism distinguishes two financing tracks that are often conflated. Operational financing covers staff, premises, logistics, training, and other recurring costs of the hub and the spokes. Device financing covers acquisition, refurbishment, maintenance, and replacement of the AT itself. We argue that the two tracks should be governed separately, because they answer to different fiscal logics. Operational expenditure is largely predictable and suited to budget-line financing, while device expenditure is volume-driven and benefits from framework-agreement smoothing. As Tay-Teo et al. (7) point out, the misalignment of financing instruments and expenditure profiles is one of the most common system-level pathologies in this field.

For operational financing we accommodate two options drawn from the comparative cases. A budget-based option treats the hub and spokes as line-item institutions whose salaries, contributions, and material costs are met from the central budget, possibly supplemented by subnational contributions for local material costs. This option, derived from the Norwegian model, offers predictability but limited service-mix flexibility. A grant-based option, derived from the Emilia-Romagna model, combines a non-purpose grant covering the baseline of operational expenses with project-based dedicated grants tied to defined service categories and minimum service thresholds (22). Grant-based financing creates incentives for productivity and supports incremental service expansion beyond the originating sector, but it presupposes administrative capacity to monitor service delivery against contractual standards. The model permits a transition from budget-based to grant-based logic as administrative capacity matures.

For device financing we are more prescriptive. Device financing should be centralised and pooled regardless of which operational option is chosen. The argument is twofold. First, pooling absorbs idiosyncratic local demand variation, which eliminates the situations in which a user's territorial origin determines whether a needed device is available. Second, only a centrally pooled budget can underwrite framework agreements at the volumes required to negotiate competitive prices, the very mechanism through which the market-shaping instruments described in Section 3.2.3.3 operate. Local self-governments retain financing roles for transport, accompanying-person costs, and other context-specific expenses that are most efficiently administered locally.

##### 3.2.3.3 Market-shaping mechanism

**Figure 3:**
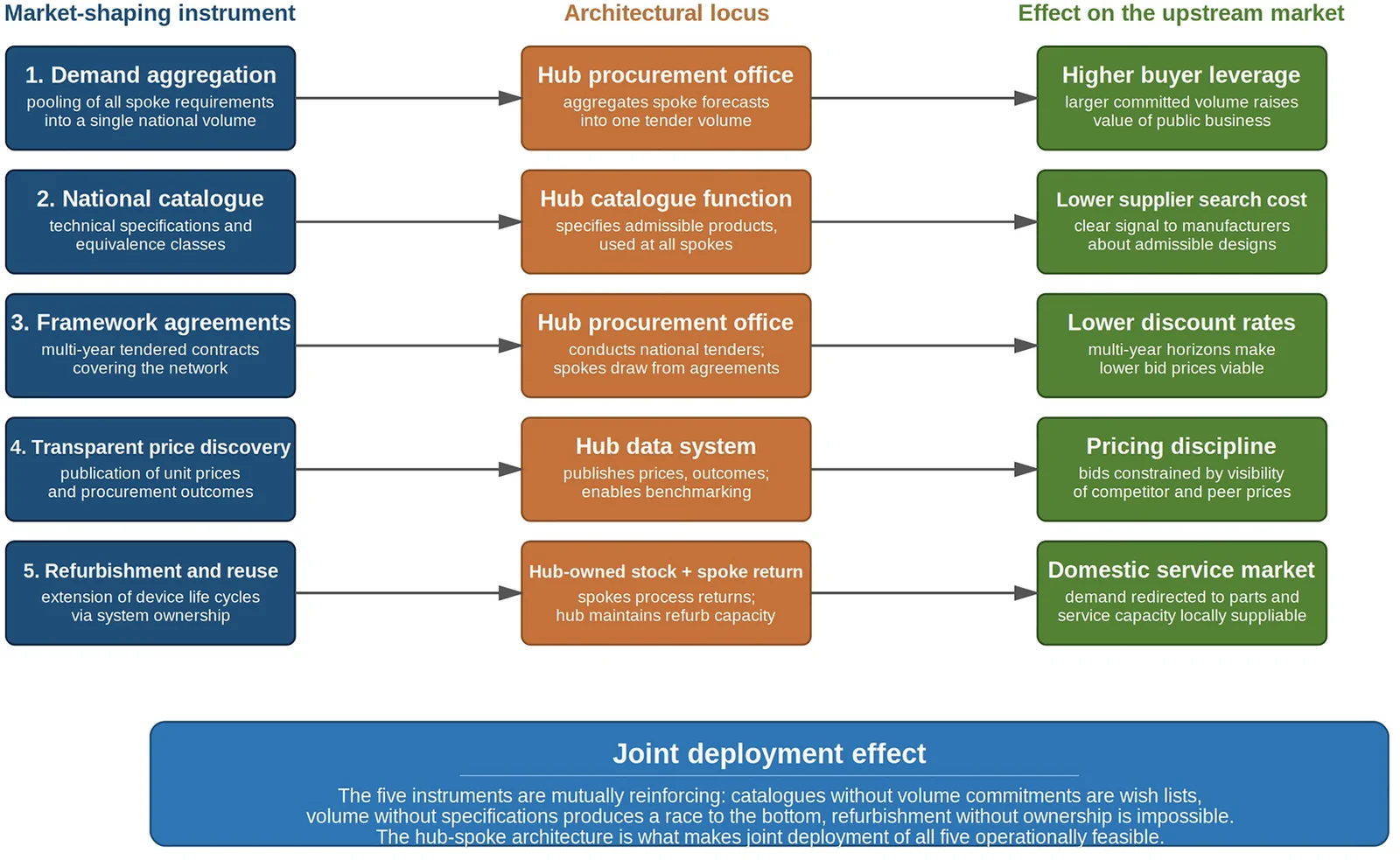
Five market-shaping instruments and their operationalisation through the hub-spoke architecture. Five market-shaping instruments and their operationalisation through the hub-spoke architecture. Each row identifies one instrument (demand aggregation, national catalogue, framework agreements, transparent price discovery, refurbishment and reuse), the architectural locus that operationalises it, and the resulting effect on the upstream market. The instruments are mutually reinforcing – catalogues without volume commitments are wish lists, volume without specifications produces a race to the bottom, refurbishment without ownership is impossible. The hub-spoke architecture is what makes joint deployment of all five operationally feasible.

The market-shaping mechanism describes how the system's purchasing behaviour shapes the upstream market for assistive products. The effects described in this section are the outcomes the model is designed to produce. They follow from the logic of the architecture and from evidence on comparable instruments in global health markets, but they have not yet been directly demonstrated for AT, and are therefore best read as hypotheses that the model generates and that future implementation can test. Five instruments operate jointly, mirroring the toolkit established in vaccine and other global health markets (5, 6), and summarised with their architectural locus and upstream effect in Figure 3. Demand aggregation pools the requirements of all spokes into a single national volume, which is expected to raise the value of public-sector business for suppliers and increases the buyer's leverage at the negotiating table. The national catalogue defines admissible product categories, technical specifications, and equivalence classes, signalling to manufacturers and distributors which product designs will reach the public market and reducing the search costs that suppliers face when assessing whether to enter. Framework agreements concluded through competitive tender translate that signal into committed volumes over multi-year horizons, which should lower the effective discount rate at which suppliers value the public market and thereby reduce prices. Transparent price discovery, achieved through publication of unit prices and procurement outcomes, is expected to discipline pricing and support cross-jurisdictional benchmarking. Refurbishment and reuse, finally, are designed to extend product life cycles, redirect a portion of demand from new acquisition to maintenance and parts, and create a secondary market for service capacity that can support domestic firms which may not be competitive in primary device manufacturing.

The five instruments are mutually reinforcing. A catalogue without volume commitments is only a wish list. Volume commitments without specifications produce a race to the bottom on quality. Price transparency without a catalogue is uninterpretable. Refurbishment without ownership is impossible. The hub-spoke architecture is what makes joint deployment of all five instruments operationally feasible. The hub holds procurement, catalogue, ownership, and price-disclosure functions; the spokes maintain stock, conduct refurbishment-eligible returns, and provide the service capacity that absorbs refurbished devices back into use. The Norwegian system, where ownership-based refurbishment accounts for 30% of AT expenditure (8), is the empirical demonstration that joint deployment is achievable and that its fiscal returns are non-trivial. Norway and Italy demonstrate market-shaping implicitly. What we add here is to make it explicit, so that transitioning systems and LMICs can deploy it deliberately and design the institutional architecture around it from the start, rather than retrofitting it onto a system designed for service delivery alone.

#### 3.2.4 Implications for trade and supply chains

The model has direct implications for international trade and supply chain organisation. When the hub aggregates national demand into multi-year framework agreements, it becomes a credible counterparty for international suppliers and a reference point for harmonised technical specifications. Specifications harmonised across hubs in different countries, for instance through regional catalogues developed under organisations such as the European Union, CEFTA, or other regional economic groupings, can reduce the transaction costs that suppliers face when entering small national markets, with potential effects on prices and product variety in countries that individually lack market scale. Use of the Harmonized System and other international classification frameworks at the catalogue level facilitates this harmonisation and supports tariff and tax coordination, particularly in regions where AT products are not separately classified and therefore default to higher general tariff lines.

Refurbishment-based ownership has additional trade-policy implications. Refurbishment extends product life cycles within the public system. As a result, it reduces import dependency at the margin and creates demand for parts and service capacity that domestic firms can supply even if they cannot compete in primary manufacturing. The result is a layered supply structure in which primary devices may continue to be imported, while a domestic service market emerges around them, with positive employment, technology-transfer, and quality effects. Norway's approximately 30% refurbishment share illustrates the steady-state magnitudes involved (8). For LMICs and middle-income countries, where local production may not be feasible at the device level but is feasible at the service level, the refurbishment loop is therefore not only an internal efficiency mechanism but also a tool for nascent industrial development. Regional procurement integration, whereby several neighbouring countries jointly tender on common framework agreements, is a further extension of the same logic and is, in principle, accessible to countries that share regulatory and trade frameworks.

### 3.3 Application: feasibility study in Serbia

Serbia is a representative transitioning context for testing the model. The country has territorial inequalities pronounced enough to expose any reliance on local financing, an institutional landscape that includes thirteen Resource Centres established under the Law on Foundations of the Education System, and a multi-source AT financing arrangement that is currently insufficient and inconsistent. Serbia is also a candidate country for European Union accession, with public procurement legislation progressively harmonised with EU directives, and a participant in regional trade arrangements that in principle enable joint procurement at the regional level. Our applied feasibility study assessed the operational, legal, and financial conditions under which the model could be implemented.

#### 3.3.1 Current AT landscape

AT provision in Serbia operates through a “mitigated medical model” in which prescribed diagnoses determine eligibility, with health insurance covering most mobility, vision, hearing, and speech-related devices. Educational AT is partially financed through the inclusive education framework, with the central government budget covering preschool programmes for children with disabilities and salaries of staff in primary and secondary schools for students with disabilities. Local self-governments are responsible for additional support recommended by Intersectoral Committees, but the legal basis for this obligation is fragmented across several articles of the Law on Foundations of the Education System and is interpreted heterogeneously across municipalities. The result is that the level of AT support a child receives is, in practice, strongly conditioned by municipal fiscal capacity rather than by need.

From a market-shaping perspective the current Serbian configuration activates few of the instruments identified in Section 3.2.3.3. There is no integrated national AT catalogue across sectors. Procurement is fragmented across health insurance funds, individual schools, and local self-government budgets. Framework agreements at national scale are absent. Refurbishment is negligible. The thirteen Resource Centres established since 2021 are an embryonic spoke network. They already perform assessment, training, and partial provision functions, and have established working relationships with educational institutions, healthcare providers, and civil society. What is missing is the hub.

#### 3.3.2 Operationalising the modified model

Our feasibility study proposes the establishment of a National Centre for Assistive Technologies (NCAT) as the hub of the system, supported by the existing thirteen Resource Centres operating as spokes. We assessed two legal pathways. The first amends the Law on Foundations of the Education System to introduce an explicit article defining NCAT, its role, and its relationship to the Resource Centres. The second uses an existing provision of the same law to designate one Resource Centre as an institution of special interest to the Republic of Serbia, which guarantees national-budget financing without legislative amendment. Both options are technically viable. We judged the second as more feasible in the short term, because it avoids the timeline and political risk of legislative amendment, while the first offers a clearer long-term institutional identity. We recommend transforming an existing Resource Centre into the hub, rather than constructing a new institution, drawing explicitly on the Armenian precedent of repurposing existing infrastructure and staff.

The functional split between NCAT and the Resource Centres in the proposed Serbian implementation maps directly onto the model. NCAT is to hold the AT catalogue, conduct centralised tenders under Serbia's public procurement legislation (now substantially aligned with EU directives, including European-level publication of major tenders), conclude framework agreements with vendors, own the devices, operate the refurbishment programme, maintain the data system, and provide advanced assessment for complex cases. The Resource Centres are to perform routine assessment, issue and personalise devices from regional stock, train users and educators, maintain devices, conduct follow-up, and coordinate with Intersectoral Committees and schools. The role of Intersectoral Committees is reinforced and clarified: their assessments must draw on Resource Centre input where AT is at issue, which addresses the inconsistency in current Intersectoral Committee practice.

#### 3.3.3 Financial architecture

The financial architecture combines elements of both operational financing options. In the near term, operational financing follows the budget-based logic. NCAT and Resource Centre salaries, contributions, and material costs are financed from the central government budget; local self-governments contribute the local material costs and the transport costs associated with services delivered to their residents. In the medium term, the architecture is to evolve toward a grant-based logic modelled on Emilia-Romagna, with three-year contracts between the Ministry of Education and the Resource Centres, distinguishing non-purpose grants from project-based dedicated grants tied to defined service categories such as assessments, training, and educational support (22).

Device financing is centralised. NCAT conducts national tenders, concludes framework agreements, owns the devices, and finances acquisition, refurbishment, and insurance from a dedicated budget line within the Ministry of Education. A transitional arrangement allows the Ministry of Education itself to perform procurement on NCAT's behalf during the start-up period, which ensures that supply continuity is not contingent on the completion of the institutional transformation. The architecture deliberately decouples device financing from local self-government budgets. This is designed to addresses the equity problem that motivates the model and to create the volume base on which the market-shaping instruments described in Section 3.2.3.3 depend.

#### 3.3.4 Implementation pathway

Our Serbian feasibility study identifies three sequencing principles relevant for any implementation of the model in a transitioning context. First, the existing Resource Centre with the deepest infrastructure and most established stakeholder relationships should be transformed first, which provides operational continuity during a phase in which the rest of the network is not yet aligned. Second, education should be the first sector covered, as it is institutionally simpler than the multi-sector environment and aligns with the existing Resource Centre mandate. Expansion to health, employment, and elderly care should follow as administrative capacity matures, and only after the basic operational and financial mechanisms are functioning. Third, the data system should be established at the outset, even before steady-state procurement volumes are reached, because retrospective data reconstruction is substantially more costly than prospective collection. These sequencing principles are generalisable. They describe the order in which the components of the model can be assembled in any country starting from a partial system.

## 4 Discussion

The modified hybrid hub-spoke model articulated in Section 3.2 is a synthesis rather than an invention. Each of its components exists somewhere in the comparative cases. What we contribute here is the specification of how the components fit together, which combinations produce mutually reinforcing effects, how the system can be assembled in stages, and how its market-shaping functions can be activated deliberately rather than incidentally.

### 4.1 Theoretical contribution

Our model contributes to three intersecting bodies of literature. With respect to AT systems, it advances beyond the dominant centralised-decentralised dichotomy by treating governance as a function-by-function question. The dichotomy is misleading, because the same system can be centralised on one dimension, such as procurement, and decentralised on another, such as assessment or follow-up. The productive question is which functions belong on which side. Our four-fold modification disaggregates the functions sufficiently to make this question tractable. In particular, the explicit treatment of refurbishment as a hub-side function and of follow-up as a spoke-side function resolves a tension that often goes unresolved in conventional hub-spoke designs (13).

With respect to hub-spoke organisation more generally, our model extends the canonical template, which has been formulated principally as a routing or service-delivery problem (13, 15), to incorporate market-shaping functions and ownership-based refurbishment loops as constitutive elements of the design. We also engage the strategic-management literature on hub-spoke games (27) by adopting an intermediate position between single-issue authority and multi-issue negotiation. Decisions are made centrally where coordination across the network delivers the largest gains—procurement, the catalogue, and technical standards. Decisions are negotiated with the spokes where local variation matters most—service mix, training priorities, and coordination with local partners. The integrated practice unit logic articulated in healthcare strategy (14) provides a parallel justification for this architecture.

With respect to global health market-shaping, we extend instruments developed for vaccines and other essential health commodities (5–7, 28) to assistive technology, while specifying the institutional architecture through which the instruments can be operationalised. To our knowledge, this is the first systematic articulation of what an AT system optimised for market-shaping looks like at the country level.

### 4.2 Findings in relation to existing literature

Our findings reinforce, qualify, and in places extend several streams of existing research. The classical decentralisation literature has long recognised that subnational delivery without compensating central financing tends to amplify rather than reduce horizontal inequalities (11, 12, 25), particularly in low- and middle-income contexts where subnational fiscal capacity is highly variable. The Serbian case examined here is consistent with this pattern. Under the current arrangement, the level of AT support a child receives is strongly conditioned by municipal fiscal capacity rather than by need. The model's prescription of centralised device financing combined with operationally distributed delivery follows directly from this observation. It also accords with the recommendation by Tay-Teo et al. (7) that financing instruments must be matched to expenditure profiles, with pooled public funds particularly suited to volume-driven device acquisition.

The comparative literature on Nordic AT systems documents a consistent pattern in which centralised procurement, ownership, and refurbishment co-occur (8, 20). Our findings confirm this co-occurrence and identify it as a causal cluster rather than a coincidence. The three features are mutually enabling, and the absence of any, tends to undermine the others. The Italian case provides an important counterpoint. Substantial market-shaping leverage can be exercised through national catalogues even where procurement is regional, provided the catalogue function is genuinely national. This finding qualifies the inference that centralisation is required across the board, and it supports our function-by-function disaggregation.

Comparative European studies of AT access and service delivery have repeatedly identified procurement and supply chain organisation as systemic determinants of access, alongside financing and human resources (2, 18, 20). The 2018 GREAT summit position paper similarly identified procurement and supply systems as among the most consequential leverage points for system change (28). What we add here is the operational detail—how a national institutional architecture can activate these leverage points in concert. Our findings on Armenia and Ukraine illustrate that institutional fragmentation is the principal binding constraint on market-shaping in evolving systems, even where political will and legal mandates are present.

### 4.3 Policy implications and transferability

Three policy implications follow. First, for transitioning national systems, the model can be built up gradually. Components can be assembled sequentially. A national AT catalogue is feasible without immediate procurement centralisation. Centralised procurement can be introduced before refurbishment capacity is fully established. Data systems can be initialised before steady-state volumes are reached. The Serbian application illustrates this graduated logic concretely. The implication for policymakers is that the absence of one component is not a reason to defer establishing the others.

Second, the financial architecture has implications beyond AT. The distinction between operational and device-level financing, with different financing logics applicable to each, is generalisable to other social-service domains that combine recurring institutional operation with volume-driven commodity procurement, children's social care, certain rehabilitation services, school nutrition, and personal protective equipment systems among them. The principle that equity-relevant commodities should be centrally pooled, while operational financing can vary in its admixture of central and local sources, may apply more broadly than the AT context that motivates it here.

Third, the implications for LMICs and regional trade integration are significant. Many LMICs face thinner national markets than even small European countries and individually lack the scale at which framework-agreement procurement secures competitive prices. Two responses are available within the model. The first is regional integration of catalogues and procurement, whereby several countries jointly tender on common specifications and split delivery across national hubs. This is in principle feasible within trade and economic groupings such as CEFTA in the Western Balkans, EAC in East Africa, or ASEAN in Southeast Asia, and it would extend to AT the joint-procurement logic already operating in vaccines and other health commodities at supranational level (5, 6). The second is the deliberate use of refurbishment to substitute domestic service capacity for imported volumes. This is feasible in the short run even where local manufacturing is not, and it supports gradual technology transfer toward more capability-intensive activities. Both responses are accessible to countries that adopt the hub-spoke architecture and apply its market-shaping logic from the design stage.

These implications converge on a broader point about the framing of AT systems. The market-shaping mechanism reframes AT systems as instruments of trade and industrial policy in addition to social policy. We do not mean to suggest that public AT systems should pursue industrial objectives at the expense of users. Systems designed without attention to their market footprint, however, forgo a productive lever, particularly in small national markets where the public system is necessarily an anchor purchaser. The Norwegian and Italian cases show two contrasting versions of this mechanism. Norway uses procurement aggregation to disciplinary effect, while Emilia-Romagna uses contractual specialisation between public and non-governmental actors to support a service market. Transitioning systems can choose between these patterns, or combine them, deliberately.

### 4.4 Limitations and future research

Several limitations qualify the analysis. The empirical base of four comparative cases supports identification of structural patterns but does not permit statistical generalisation. The cases were selected purposively rather than as a representative sample, and all four are European, which limits transferability to lower-income settings without further empirical work. The model presented in Section 3.2 is a normative design. It specifies what a national AT system optimised for equity, efficiency, and market-shaping should look like, and how its components should fit together. It is derived from patterns observed in existing systems, and its logic is internally consistent, but it has not yet been implemented and evaluated in that combined form. The Serbian application in Section 3.3 is likewise a design and feasibility study rather than an implementation outcome. It shows that the model is administratively, legally, and financially plausible in a transitioning national setting, but it does not demonstrate that the predicted equity, efficiency, and market-shaping gains have materialised. That test can only come from longitudinal evaluation once the model is in operation, which we identify below as the first priority for future research. Cost estimates derived from the feasibility study rely on benchmarks from Italian and Norwegian sources where Serbian primary data are unavailable, particularly with respect to refurbishment economics in steady state. The model is most fully developed for the education sector and requires further articulation for cross-sectoral expansion to health, employment, and elderly care, where additional ministerial actors complicate the institutional design. Finally, the trade-policy dimension is treated here at the level of mechanism rather than detailed policy. A fuller analysis would engage tariff schedules, the Harmonized System classification of assistive products, and the value-added tax treatment of AT in specific national settings.

A further set of limitations concerns the risks that the design itself introduces, which the analysis so far has treated too lightly by presenting centralisation as nearly costless. Five failure modes deserve particular attention. First, procurement capture: a small number of pre-qualified suppliers may come to dominate framework agreements and erode competitive pressure over time, a risk that periodic re-tendering, transparent selection criteria, and rotation of technical evaluators can mitigate but not eliminate. Second, supplier lock-in: reliance on a single vendor for a device category can become system-critical, particularly for advanced or specialised devices, which makes multi-vendor sourcing and interoperability requirements in the catalogue important safeguards. Third, the start-up costs of refurbishment: the depots, logistics, technical staff, and stock-management systems that the refurbishment loop requires demand significant upfront investment, so that in the transition period refurbishment adds cost rather than saving it, and the fiscal returns observed in mature systems appear only once the loop is running at scale. Fourth, hub capacity risk: the central hub can become an operational bottleneck, especially during start-up, which argues for graduated implementation and for legal continuity arrangements that preserve service delivery if hub capacity is temporarily insufficient. Fifth, political and institutional risk: a single national institution is vulnerable to shifts in political priorities, ministerial reorganisation, and budgetary retrenchment, which makes a durable legal basis, of the kind discussed for Serbia in Section 3.3.2, a condition for resilience rather than a formality. None of these risks is disqualifying, but a system designed without regard to them is unlikely to realise the gains the model predicts.

Future research should pursue five directions. First, longitudinal evaluation of the Serbian implementation, once initiated, will permit empirical testing of the predicted equity, efficiency, and market-shaping effects. Second, comparative evaluation of budget-based and grant-based operational financing arrangements would clarify the conditions under which each performs better. Third, empirical measurement of market-shaping effects of public AT procurement on prices, quality, and supply diversity in small national and regional markets is needed; the literature on health commodity market-shaping (5, 6) provides methodological templates that can be adapted. Fourth, the model's extension to LMICs and to regional procurement arrangements deserves direct empirical attention, including engagement with the policy and trade dimensions only briefly addressed here. Fifth, future work should incorporate the perspectives of AT users and people with lived experience of AT provision. The model developed here operates at the system and organisational level, and its predicted equity gains ultimately have to be experienced by users themselves. User-centred evaluation, including qualitative research with children, adults with disabilities, families, and front-line practitioners, is essential to validate whether the system-level design translates into meaningful improvements in access, choice, and daily use of assistive products.

The transferability of the model rests on relatively few preconditions. National legislation must permit centralised procurement and a national AT catalogue. A lead institution must be designated with operational authority across procurement, catalogue, and standards. A network of regional facilities, however small, must exist or be feasible to establish. Operational financing must be available, even if limited. Resources for device acquisition must be poolable into a single national budget line. Where these preconditions are met, as they are with varying degrees of effort in most countries of Central and Eastern Europe, the Western Balkans, and many middle-income countries beyond Europe, the model is implementable. Where they are not, our comparative analysis identifies which precondition is binding, which is itself a useful diagnostic.

## 5 Conclusion

The modified hybrid hub-spoke model articulated in this paper offers a transferable institutional architecture for AT delivery, optimised for equity, efficiency, and market-shaping. Our principal contribution is to specify how four design components, integrated refurbishment, catalogue- and framework-agreement-based procurement, graduated assessment, and dual-track financing, combine into a coherent system that activates market-shaping instruments deliberately. With current AT coverage rates well below what is required by the UN Convention on the Rights of Persons with Disabilities, the question of how to deliver AT equitably and at scale will only become more pressing in the years ahead. We offer the model as one durable answer to that question. It is accessible to transitioning systems and LMICs in a graduated form, and it is extensible to regional procurement integration where national markets are too thin to be shaped on their own. Finally, the experiences of Norway and Italy in particular show that successful AT systems are built incrementally, over years rather than months. Countries that begin now, even with limited resources, can realistically expect to see results within a planning horizon that is shorter than they may assume.

## Data Availability

The original contributions presented in the study are included in the article/Supplementary Material, further inquiries can be directed to the corresponding author.
